# Enhanced phosphate removal by zero valent iron activated through oxidants from water: batch and breakthrough experiments

**DOI:** 10.1039/d1ra05664f

**Published:** 2021-12-15

**Authors:** Weilong Zeng, Bing Li, Xueying Lin, Sihao Lv, Weizhao Yin, Ping Li, Xiangyu Zheng, Jinhua Wu

**Affiliations:** School of Environment and Energy, South China University of Technology Guangzhou 510006 China jinhuawu@scut.edu.cn + 86 20 39380569 + 86 20 39380569; School of Light Industry and Materials, Guangdong Polytechnic Foshan 528041 China; College of Chemistry and Environmental Engineering, Dongguan University of Technology Dongguan 523808 China; School of Environment, Jinan University Guangzhou 510632 China; The Key Laboratory of Pollution Control and Ecosystem Restoration in Industry Clusters, Ministry of Education Guangzhou 510006 China; The Key Laboratory of Environmental Protection and Eco-Remediation of Guangdong Regular Higher Education Institutions Guangzhou 510006 China

## Abstract

In this study, oxidants including hydrogen peroxide (H_2_O_2_), hypochlorite (ClO^−^) and persulfate (S_2_O_8_^2−^) were employed to promote zero-valent iron (ZVI) corrosion and enhance phosphate (P) removal from water through batch and breakthrough experiments. Characterization results indicated that the addition of oxidant can cause large-scale corrosion of the iron surface. This subsequently generates more iron ions and active minerals, resulting in a large number of reaction-adsorption sites for P removal. Therefore, compared with the ZVI alone system (29.4%), the removal efficiency of P by oxidant/ZVI system (H_2_O_2_ : ClO^−^ : S_2_O_8_^2−^ = 33.2% : 54% : 67.1%) was improved. For the oxidant/ZVI system, H_2_O_2_ can promote the corrosion of ZVI to a certain extent. However, the solution pH could be increased during the corrosion process. This leads to inhibition of P removal performance by the H_2_O_2_/ZVI system, which only increased by 12.9% to 33.2%. The reaction between NaClO and ZVI consumes less H^+^, and the reaction product Cl^−^ can pierce the passivation layer on the surface of the ZVI through the pitting effect. As such, the NaClO/ZVI system attained a 54% P removal rate. Compared with H_2_O_2_ and NaClO, a better P removal effect of about 67.1% can be achieved by using Na_2_S_2_O_8_, since the oxidation corrosion process of Na_2_S_2_O_8_ does not consume H^+^, and it also has the strongest oxidizing properties. Furthermore, an appropriate increase in oxidant dosing (0.1–2 mM) could improve the efficiency at which of P is removed. Five batch cycle experiments showed that the oxidant/ZVI system has a higher removal capacity and longer life-span. In the long-term column running, the P removal capacity and operation life of the NaClO/ZVI column are 9.6 times and 3.2 times higher than that of the ZVI column, respectively. This work demonstrates that an oxidant/ZVI system can be an efficient method for P removal in water, which also provides a new idea for solving the problem of ZVI corrosion passivation.

## Abstract 2

This model is used to illustrate the enhanced P removal by oxidant stimulated ZVI.

## Graphical abstract

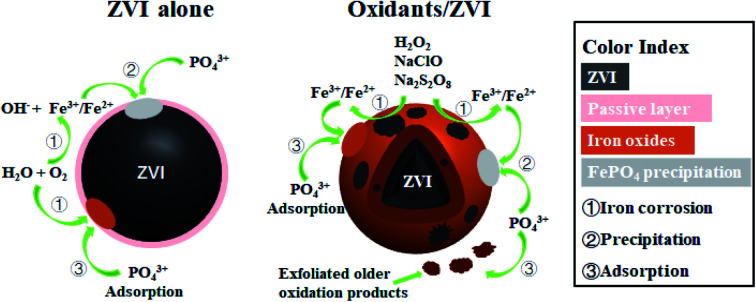

## 1. Introduction

Excessive release of nutrients such as nitrogen and phosphorus into natural water bodies *via* human activities usually leads to water eutrophication.^1–4^ This causes serious water pollution problems, such as the growth of cyanobacteria and associated oxygen depletion, algal blooms, massive amounts of fish deaths and disruption of aquatic ecosystems.^5,6^ Therefore, it is necessary to be addressed urgently. Previous studies have shown that nitrogen can circulate between the gas and liquid phase through biological nitrification and denitrification reactions,^6–8^ so controlling phosphorus is a key factor in reducing the eutrophication of fresh water.^9–12^ It has been reported that a phosphorus concentration in excess of 0.02 mg L^−1^ can cause eutrophication.^13^ Therefore, the discharge standard for municipal wastewater treatment plants in China have a maximum allowable concentration of phosphorus in wastewater that is less than 1 mg L^−1^.^14^ Recently, despite efforts to reduce the quantities of phosphorus from pollution sources, phosphorus contamination in water is still a major environmental issue.^6^ It is therefore still incredibly important to develop a novel and efficient technique for removing phosphorus.

To date, different technologies, such as chemical precipitation,^15^ biological treatment,^16,17^ membrane filtration,^18^ and adsorption^19,20^ have been developed for wastewater phosphate removal. However, each technique discussed above has its own limitations. For example, the chemical precipitation method requires the addition of many chemical reagents and produces a large amount of sludge, which can cause secondary pollution and increase treatment costs.^21,22^ Biological treatment complex and inefficient,^23^ and the resulting treated domestic sewage often fails to meet the strict phosphorus emission standards. Chemical precipitation methods are often required to remove phosphorus in the tail water.^24,25^ Membrane filtration requires regular maintenance to avoid membrane fouling, resulting in high costs and complex operation.^26–28^ Compared with the above methods, the adsorption method has been recommended because of its relatively high efficiency, simple operation method and comparatively low cost.^29^ So far, several adsorbents have been reported for wastewater phosphate removal, such as slag,^30^ clay,^31^ fly ash,^32^ zeolite^33^ and ZVI.^34^ Among them, ZVI has attracted a lot attention as an adsorbent for removing water pollutants through physical and chemical adsorption, such as electrostatic adsorption, complexation and co-precipitation.

Recently, ZVI has been used as a highly efficient medium for removing chlorinated organic compounds,^35^ nitroaromatics,^36^ arsenic^37^ and nitrate^38^ in water due to its reduction effect. In addition, it can also remove heavy metals such as Cd, Cr and Pb in water using iron oxides formed on the water's surface with environmental media (O_2_ and H_2_O).^39,40^ However, regardless of the mechanism, the basis on which pollutants are removed is the surface corrosion of ZVI.^37^ The efficient use of ZVI to removal P still faces some challenges. One serious issue is that during the production and storage of ZVI, a layer of oxides will be formed on the surface, which makes it difficult to corrode.^41^ Moreover, phosphate reacts with iron ions, the corrosion product of ZVI, to generate dense ferric phosphate compounds. These compounds cover the iron surface to form a passive layer, preventing further corrosion of ZVI.^42^ To date, there are various methods and techniques for improving ZVI reactivity such as acid washing,^43^ H_2_-reduction pretreatment,^44^ weak magnetic fields,^45^ ZVI-based bimetals,^46^ and nanosized ZVI (nZVI).^47^ However, these techniques are always complicated, expensive and toxic to the environment.

Many studies reported that researchers tried to add a small amount of oxidant, including NaClO, KMnO_4_ and H_2_O_2_ into the ZVI decontamination system.^48^ These oxidants can pierce the oxide film on the surface of ZVI and accelerate the corrosion of ZVI. This significantly improves the reduction rate of nitrate and the removal performance of selenite and heavy metals (Cd, Hg, Sb) in water.^37,42,49,50^ However, these studies did not discuss the mechanism of different oxidants promoting ZVI corrosion. In this study, using phosphate as the target pollutant, the effect of adding different oxidants (H_2_O_2_, NaClO, Na_2_S_2_O_8_) on the removal of phosphate by ZVI was compared. The influence of the amount of oxidant added on the strength of its P removal effect was studied. In order to verify that the oxidant can continuously drive ZVI corrosion, five batch cycle and long-term column running experiments were conducted. Finally, scanning electron microscopy (SEM), X-ray diffraction (XRD) and other characterization methods were employed. The impact on the surface morphology of ZVI before and after the addition of oxidants and the formation of secondary minerals on the surface are analyzed. Besides, the possible mechanism of different oxidants is explored to promote the removal of phosphate by ZVI.

## 2. Materials and methods

### 2.1 Chemicals

All reagents used in the experiment, including NaClO (Cl ≥ 7.5%), 30% H_2_O_2_, and Na_2_S_2_O_8_ were purchased from Guangzhou Chemical Reagent Factory. Chemical reagents used in this work are all analytical grade, and the solutions are all prepared with deionized water. ZVI particle size for batch experiments was 0.15 mm, purchased from Shanghai A Latin (Shanghai). The ZVI particle size used in the column experiment was 1 mm, and purchased from Shandong Group Co., Ltd. Quartz sand (1 mm) obtained from Shilin Building Materials Company for dispersing the iron and adjusting the porosity of the column reactors. Prior to use, the sand was soaked in 10 mg P/L phosphate solution for 24 h. The phosphate stock solution (50 mg P/L) was prepared and diluted for the experiments. All glassware is soaked in 15% nitric acid before being used and washed with tap water and deionized water.

### 2.2 Batch experiment

Batch tests were conducted in a 300 mL conical flask by adding an equivalent phosphate solution (10 mg P/L, 250 mL) and 0.25 mM of oxidants (H_2_O_2_, NaClO and Na_2_S_2_O_8_). The initial pH value of 7.0 ± 0.1 was obtained by regulating the amount of 0.1 M HCl and NaOH added. The flask was then dosed with 0.25 g ZVI. The Erlenmeyer flask was then covered with a rubber stopper and placed in a shaking shaker. The reaction was carried out at room temperature of 25 ± 2 °C and a rotation speed of 35 rpm. During sampling, 3 mL solution samples were collected, and filtered with a 0.45 μm syringe for further analysis. For comparison, a set of systems without oxidizing agents was made as a control. By changing the dosage of oxidant (0.1–2 mM), the effect of adding oxidant on the removal of phosphate by ZVI was investigated. In order to investigate the sustainability of oxidant/ZVI coupling in removing P, 5 cycles of experiments were carried out. Each experiment was conducted three times and the average value was calculated.

### 2.3 Column experiment

**Fig. 1:**
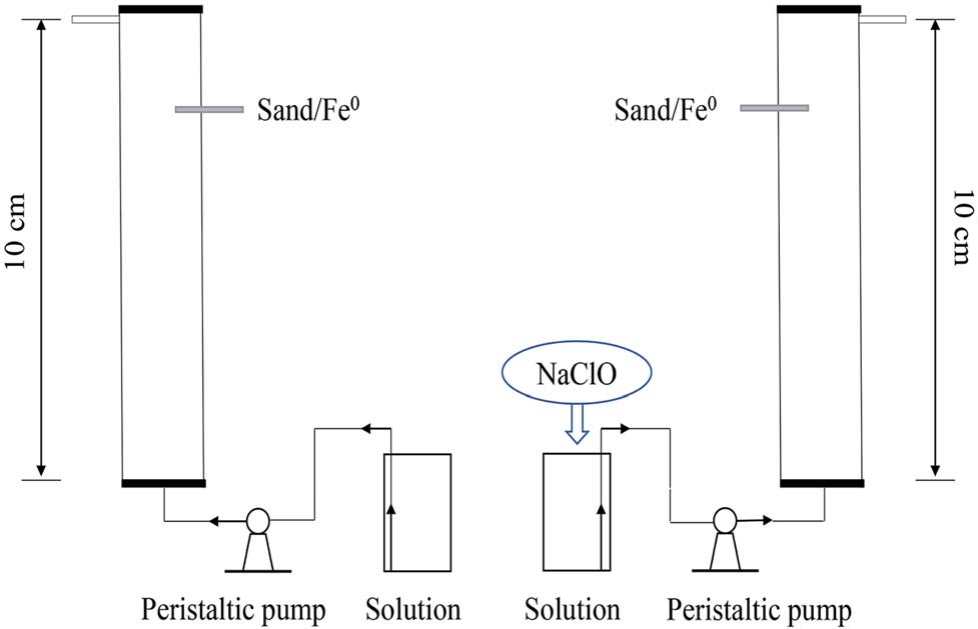
Fixed-bed purification device.

Column experiments were conducted in PVC pipe columns (1.7 cm ID × 10 cm L). As described in Fig. 1, two columns were packed with sand and iron particles (iron packing density 0.44 g cm^−3^, pore volume 11 ± 0.5 cm^3^ per PV, porosity 0.48 ± 0.02). NaClO (0.5 mM) was added directly into the simulated phosphate wastewater (10 mg P/L). The solution was fed into the columns from bottom to top through a peristaltic pump at a flow rate of 0.36 mL min^−1^ and the required time for 1 PV was 40 min. To create a control, no NaClO is added to the phosphate wastewater simulating influent to the other column.

### 2.4 Characterization and data analysis

The determination of phosphate adopts molybdenum antimony spectrophotometry (UV-5200, Metash, China). After the comparative experiment, all reacted iron samples were freeze-dried overnight, and then sealed and loaded under anaerobic conditions. Unreacted iron powder was prepared in the same way. The surface structure and elemental mapping of the abovementioned iron samples were analyzed by using Brunauer–Emmett–Teller (BET, NOVA4200e, America), X-ray diffraction (XRD, Empyrean Range, Netherlands) and scanning electron microscope/energy dispersive spectroscopy (SEM/EDS, ZEISS Merlin, Germany). Total Fe was detected by atomic adsorption spectroscopy (AAS, Haiguang, China). The pH values were monitored by a pH probe (SP2100, Suntex, China).

The phosphate elimination capacity of the column experiments could be calculated by eqn (1) and the method reported by Lai and Lo:^51^

1 *RC* = *C*_0_/(*M* × 1000 × *ρ*_b_)

where *C*_0_ (mg L^−1^) was the influent phosphate concentration. *M* (cm cm^−3^) was the normalized migration rate of the phosphate front at the relative concentration (*C*/*C*_0_ = 0.5), which was the migration rate (cm per PV)/the pore volume of the column (cm^3^ per PV). *ρ*_b_ (g cm^−3^) was the bulk density of the iron packed in the columns.

## 3. Results and discussion

### 3.1 Surface analysis

**Fig. 2:**
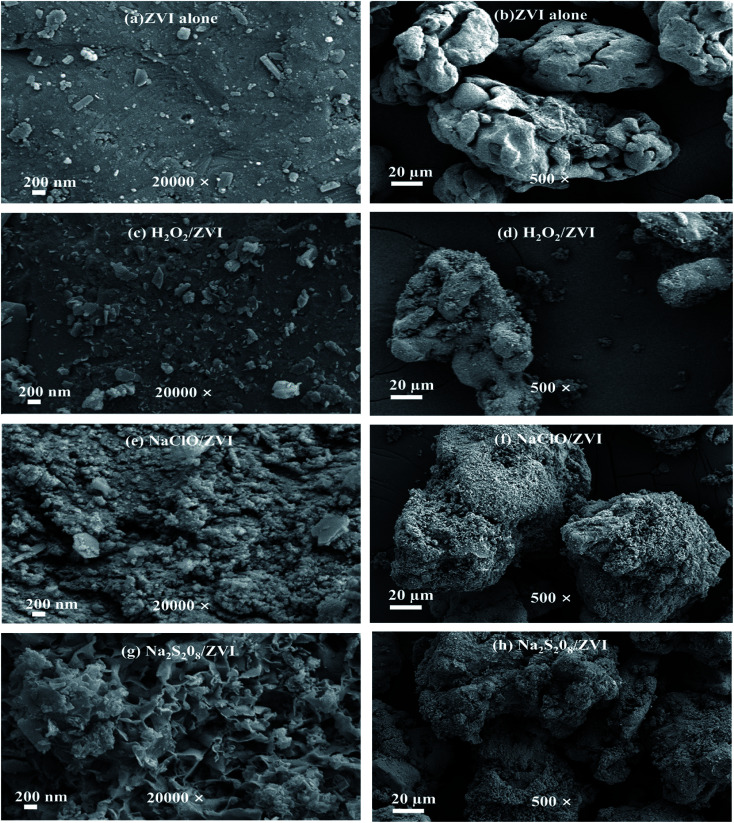
SEM image of ZVI collected after the batch test for P removal ((a and b) ZVI alone; (c and d) H_2_O_2_/ZVI; (e and f) NaClO/ZVI; (g and h) Na_2_S_2_O_8_/ZVI).

**Fig. 3:**
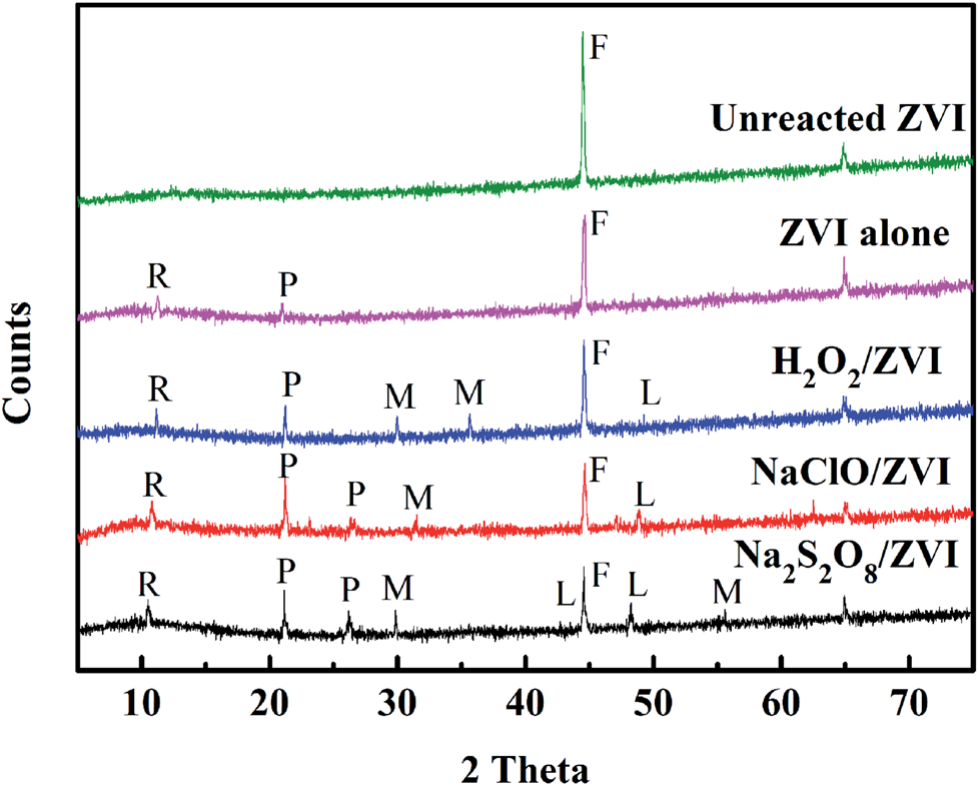
XRD results of different materials (R: iron hydrogen phosphate hydrate; P: iron phosphate; L: lepidocrocite; M: magnetite).

The surface morphologies of iron particles after reaction under different magnification levels are shown in Fig. 2. The reacted ZVI under different reaction conditions showed varying degrees of corrosion and morphological differences. In the absence of oxidants, the surface of ZVI is dense and some granular depositions are covered on the iron surfaces (Fig. 2a), due to the deposition of iron (hydrogen) oxides and ferric phosphate compounds (Fig. 3 and Table 1). In addition, at 500 times magnification, it can be seen that the degree of corrosion is low, and a considerable part of it is flat and smooth (Fig. 2b). When the ZVI is coupled with oxidants, more large-scale iron corrosion occurred on the surface of ZVI due to stimulation by oxidants. In the case of H_2_O_2_/ZVI, the degree of corrosion of the iron surface is significantly enhanced (Fig. 2d), and some micro-flakes and needle-like nano-scale crystals were observed in the SEM image (Fig. 2c). In the NaClO/ZVI system, severe oxidation corrosion can be observed on the iron surface (Fig. 2f), and the surface was covered with corrosion product clusters composed of tiny aggregates (Fig. 2e). The surface of ZVI added with Na_2_S_2_O_8_ also undergoes severe oxidative corrosion (Fig. 2h). Apart from granular substances and flaky crystals, a large number of petal-like structure minerals have also been discovered (Fig. 2g).

**Table 1:** Elemental composition of materials before and after reaction

Sample	Elements (wt%)			
	Fe	O	P	C
Unreacted ZVI	93	1.5	0.4	5.1
ZVI alone	80.74	10.63	2.03	6.6
H_2_O_2_/ZVI	72.97	16.45	2.98	7.36
NaClO/ZVI	62.21	25.08	4.12	8.59
Na_2_S_2_O_8_/ZVI	57.51	28.20	5.56	8.73

As shown in Table 1, EDS analysis revealed the elemental composition on the ZVI surface. It can be seen that the content of Fe, O, and P in the absence of oxidants were 80.74%, 10.63% and 2.03% respectively, while a much lower proportion of Fe (72.97%, 62.21% and 57.51%) and higher proportion of O (16.45%, 25.08 and 28.20%) and P (2.98%, 4.12% and 5.56%) were detected in the H_2_O_2_/ZVI, NaClO/ZVI and Na_2_S_2_O_8_/ZVI systems, respectively. These data indicated that the presence of oxidants could effectively promote the corrosion of ZVI and diverse mineral formation. As a consequence, more reaction sites were available for P adsorption.

In order to identify the nature of the iron oxides produced by ZVI surface oxidation, XRD analysis was conducted for the unreacted and reacted ZVI samples (Fig. 3). The results showed that the iron corrosion formed by the oxidant/ZVI system is more diverse than that of the unreacted and separate ZVI systems. Furthermore, multiple active iron-containing minerals such as lepidocrocite and magnetite have been discovered. In addition, compared with the ZVI alone system, the oxidant/ZVI system found more iron hydrogen phosphate hydrate and ferric phosphate compounds. These results are consistent with previous studies conducted by Sleiman^52^ and Liu.^53^ The active iron minerals attached to the ZVI surface undoubtedly increase the specific surface area of iron, and it significantly affects the removal of phosphate through various mechanisms including adsorption, precipitation and co-precipitation.^54^

### 3.2 Batch test

**Fig. 4:**
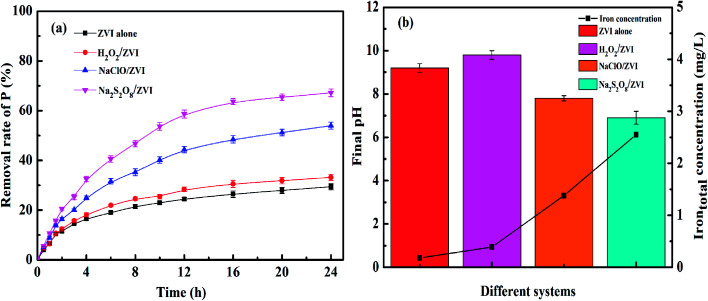
(a) P removal in different systems; (b) pH value and total iron concentration after reaction (PO_4_^3^–P = 10 mg L^−1^, ZVI = 1 g L^−1^, oxidants = 0.25 mM, initial pH = 7.0, temperature = 25 °C).

Fig. 4a illustrates the phosphate removal effect by ZVI under the addition of different oxidant conditions and pure ZVI used as control. The concentration of phosphate decreased by only 29.4% in the ZVI system alone, which is mainly ascribed to the oxidation and corrosion reaction of ZVI with oxygen in the air during the formation and storage process. A dense oxide film forms on its surface, passivating the iron surface.^41,55^ In addition, the corrosion releases iron ions, which will react with PO_4_^3−^ to produce an insoluble iron phosphate compound, which inhibits the corrosion of ZVI to a significant extent.^56^ A 33.2% phosphate removal rate in the H_2_O_2_/ZVI combined system was a slight improvement. This result was different from the results observed in the removal of heavy metals in the H_2_O_2_/ZVI system, where it could remove heavy metals as efficiently as other oxidants.^37^ This may be determined by its reaction mechanism with iron. As shown the following reactions in eqn (2) and (3).

2 ZVI + H_2_O_2_ + 6H^+^ → 2Fe^3+^ + 2H_2_O + 2H_2_

3 H_2_O_2_ + Fe^2+^ + 2H^+^ → Fe^3+^ + 2H_2_O

Compared with other oxidants, the reaction between H_2_O_2_ and ZVI requires more H^+^ to be consumed in the corrosion reaction, resulting in a higher pH value of 9.8 (Fig. 4b). Therefore, H_2_O_2_ can promote the corrosion of ZVI to a certain extent. However, as the corrosion reaction occurs, the pH in the solution increases and the corrosion of ZVI will be inhibited. Compared with the H_2_O_2_/ZVI system, the addition of NaClO showed a more significant promoting effect on the removal of phosphate by ZVI and the removal percentage reached 54.0%. This can be explained by the following two aspects. Firstly, hypochlorite has a stronger oxidation potential than O_2_ and can undergo oxidative corrosion directly on the ZVI surface, causing ferric ions to continuously form.^50^ Therefore, this generates iron oxide on the surface of ZVI to form an iron phosphate compound. Secondly, the reaction produces Cl^−^ ions which have a small radius and strong penetrating ability. This will corrode the iron oxide layer and increase the active adsorption sites.^56^ The oxidation corrosion reaction with ZVI is shown in eqn (4) and (5).

4 ClO^−^ + Fe^0^ + 2H^+^ → Fe^2+^ + Cl^−^ + H_2_O

5 2Fe^2+^ + ClO^−^ + 2H^+^ → 2Fe^3+^ + Cl^−^ + H_2_O

The Na_2_S_2_O_8_/ZVI system showed the highest level of phosphate removal, which was boosted to 67.1%. This is 1.3 times higher than that of the ZVI alone system as the oxidation of Na_2_S_2_O_8_ is the strongest among these oxidants. It can continuously produce a large amount of iron ions in the presence of ZVI. The reaction formula is shown in eqn (6) and (7). As shown in Fig. 4b, the concentration of iron ions reached a peak of 3.0 mg L^−1^. This is significantly higher than the 0.2 mg L^−1^ of the ZVI alone system, 0.4 mg L^−1^ of the H_2_O_2_/ZVI system and 1.4 mg L^−1^ of the NaClO/ZVI system. On one hand, large amounts of ferric ions use adsorption to remove phosphate by producing iron oxides such as iron hydroxide through hydrolysis. On the other hand, they directly precipitate with phosphate to produce iron phosphate compounds.^57^

6 S_2_O_8_^2−^ + Fe^0^ → Fe^2+^ + 2SO_4_^2−^

7
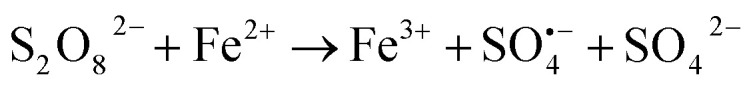


Fig. 4b describes the iron ion concentration and solution pH value in the ZVI alone system, the H_2_O_2_/ZVI system, the NaClO/ZVI system and the Na_2_S_2_O_8_/ZVI system after the reaction. It can be seen from the figure that after the reaction, the pH of the Na_2_S_2_O_8_/ZVI system solution is only 6.9, and the iron ion dissolution concentration reaches 2.55 mg L^−1^. According to reaction equations eqn (6) and (7), the oxidation and corrosion reaction between Na_2_S_2_O_8_ and ZVI does not involve H^+^, resulting in a low-pH solution. This is not conducive to the precipitation of iron ions, so a large amount of dissolved iron ions are retained in the solution. Iron ions are slowly transformed into iron (hydrogen) oxides in the subsequent ZVI H^+^-consuming corrosion reaction, which also explains why the Na_2_S_2_O_8_/ZVI system can effectively remove phosphate. The iron ion concentrations of the H_2_O_2_/ZVI and NaClO/ZVI systems are 0.39 and 1.38 mg L^−1^ respectively, while it is only 0.18 mg L^−1^ in the ZVI system alone. This shows that the iron ion concentration in the solution indirectly reflects the degree of corrosion for iron and is directly related to the efficient removal of phosphate.

### 3.3 Effect of oxidant dosage and cycling experiments

**Fig. 5:**
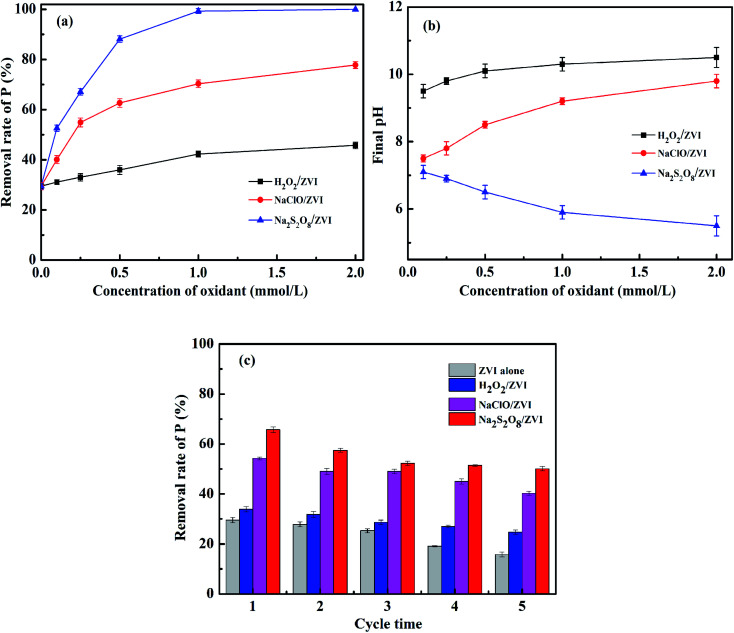
Effect of oxidant dosage on (a) P removal and (b) final pH (PO_4_^3^–P = 10 mg L^−1^, ZVI = 1 g L^−1^, initial pH = 7.0, temperature = 25 °C); (c) effect of cycling experiments on P removal (PO_4_^3^–P = 10 mg L^−1^, ZVI = 1 g L^−1^, oxidants = 0.25 mM, initial pH = 7.0, temperature = 25 °C).

The effect of oxidant dosage on phosphate removal in the oxidants/ZVI system was investigated by increasing the oxidant dosage from 0.1 to 2 mM. As shown in Fig. 5a, it can be seen that in the presence of an oxidant, the removal of P is clearly increased. This is positively correlated with the concentration of oxidant. When the concentration of oxidant increases from 0.1 to 1 mM, the removal rates of P by H_2_O_2_/ZVI and NaClO/ZVI systems were 42.3% and 70.3% respectively. These values were increased by 45.1% and 160% respectively from their original values at 0.1 mM. It can be explained that the higher the oxidant concentration, the more intense the corrosion of the ZVI surface, resulting in more ferric ions and phosphate adsorption active sites. However, when the oxidant concentration was further increased from 1 to 2 mM, the removal rate of P by H_2_O_2_/ZVI and NaClO/ZVI systems only increases by 8.3% and 10.7% respectively. A possible explanation is that the oxidant is overloaded in the system, and as a result the iron dosage becomes the rate limiting factor. In addition, ZVI corrosion promoted by excessive H_2_O_2_ and NaClO will lead to an increase in pH value. When the dosage of H_2_O_2_ and NaClO is 2 mM, the pH value of the solution after reaction is 9.8 and 10.5 (Fig. 5b) respectively. However, an excessively high pH will limit further progress of the iron corrosion reaction.^58^ For the Na_2_S_2_O_8_/ZVI system, the P removal rate increased with the increase in oxidant concentration and reaches 99.3% at 1 mM. In addition to the higher concentration of Na_2_S_2_O_8_, the fuller the corrosion of ZVI. Another important reason is that the pH of the solution after reaction gradually decreases as the concentration of Na_2_S_2_O_8_ increases. When the oxidant dosage is 1 mM, the pH of the solution after reaction is 5.9 (Fig. 5b) which is conducive to the dissolution of iron. Thus, when the concentration of Na_2_S_2_O_8_ exceeds 1 mM, the phosphate can be almost completely removed.

In order to better understand the mechanism of the oxidant promoting ZVI corrosion and its long-term effect on ZVI, we conducted five cycles of oxidant/ZVI experiments. It can be seen from Fig. 5c that after five usage cycles of the ZVI system alone, the P removal rate decreased significantly from 29.5% to 15.8%, which was a decrease of 46.4% compared to the initial cycle. This illustrates that the iron oxide and ferric phosphate compounds generated from ZVI corrosion formed a stable passivation layer that gradually clogged the reaction interface. The blockage becomes more serious as the number of cycles increases.^41^ After the oxidant/ZVI system has been used for five cycles, the removal of P by H_2_O_2_/ZVI, NaClO/ZVI, and Na_2_S_2_O_8_/ZVI systems was 24.8%, 40.3%, and 50.1% respectively, which decreased by 26.8%, 25.8% and 23.9% respectively relative to the first cycle. Compared with the ZVI alone system, this showed that the oxidant can drive the oxidation corrosion of ZVI continuously and in a stable manner. This may be due to the fact that the oxidant promotes the shedding of the older oxide layer covering the surface of ZVI.^37^ The old iron oxide layer falls off and exposes new ZVI to continue oxidative corrosion.^37^ This generates new iron (hydrogen) oxides, which increases phosphate removal efficiency, handling capacity and life-span.

### 3.4 Column study

**Fig. 6:**
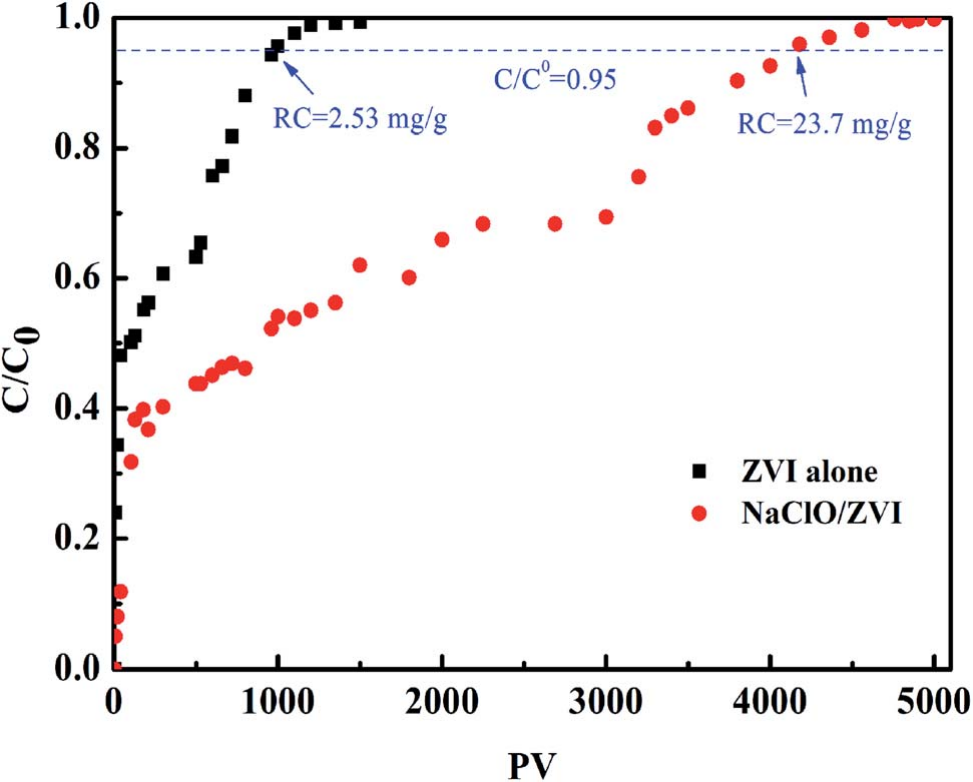
P elimination in ZVI alone and NaClO/ZVI columns (PO_4_^3−^–P = 10 mg L^−1^, oxidant = 0.5 mM, flow rate = 0.36 mL min^−1^, initial pH = 7.0, temperature = 25 °C).

The batch test demonstrated that oxidants can promote the elimination of phosphate by ZVI and could improve reliability stability and reusability during the treatment of phosphorus-containing wastewater. However, in practical applications, phosphate removal usually runs in a continuous flow state. Among the three common oxidants discussed above, NaClO is cheap and the reduction product is Cl^−^, which will not cause secondary pollution to the environment. Therefore, the NaClO/ZVI system was selected, and the ZVI alone system was used as a control, and the column was used to investigate the long-term performance of phosphate elimination under continuous flow. The break through curve is displayed in Fig. 6. In this study, a relative concentration of 0.95 (*C*/*C*_0_) was set to be the saturation point. It can be observed that phosphate removal efficiency of the column containing the ZVI alone system is much lower than that of the NaClO/ZVI system, reaching the saturation point at 970 PV, and its phosphate removal capacity is only 2.6 mg g^−1^. The corresponding phosphate migration rate reaches 0.1 cm per PV. Under aerobic conditions, the ZVI surface corrodes, producing iron oxide with FePO_4_ covering its surface. This then evolves into a thick passivation layer over time, leading to a decrease in the activity of the ZVI reaction within the column and eventually the adsorption saturation point is reached.^52^ For the NaClO/ZVI column, slow phosphate migration rate of 0.01 cm per PV is obtained. This resulted in a longer breakthrough point at 4080 PV and a higher phosphate elimination capacity of 23.7 mg g^−1^, which was 9.4 times that of the separate ZVI system column. This result was also consistent with that of batch experiments. This is mainly ascribed to the continuous dissolution of elemental iron driven by NaClO, and stable yielding of iron (hydrogen) oxides, which leads to the continuous and highly efficient removal of P. In addition, when being continuously operated, the continuous addition of NaClO can cause the older corroded layer to fall off the ZVI surface, exposing fresh ZVI which prompts the continuous generation of fresh iron oxide and hydroxide, and the continuous removal of P.^37,56^ Hence, the NaClO/ZVI column achieved a much higher P elimination capacity and had a longer life-span than the ZVI alone column.

### 3.5 Elimination mechanism

**Fig. 7:**
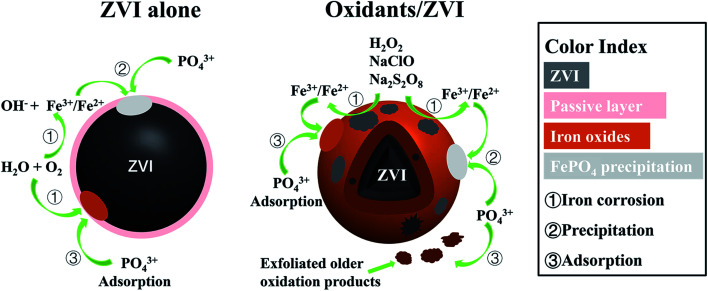
Model to illustrate the enhanced P removal by oxidant stimulated ZVI.

According to the above analysis, a possible model was proposed to illustrate the enhanced mechanism of P removal by oxidant stimulated ZVI (Fig. 7). When reacting under the condition of ZVI alone, a core–shell structure is formed on the iron surface, which is surrounded by a passivation layer, resulting in poor adsorption of P. However, when three oxidants are added, intense oxidation corrosion occurs on the iron surface, which can accelerate iron dissolution and promote the formation of an iron oxyhydroxide with high activity and adsorption properties. These include goethite, lepidocrocite and magnetite, which enhance the adsorption capacity of phosphate. Sustained and efficient removal of phosphate can at least be represented by the following eqn (8)–(11). Furthermore, the oxidant encouraged the older layer to shed and constantly form fresh iron oxides/oxyhydroxides. This further optimized the overall elimination effect of P and provided a new pathway for the removal of P.

8 Fe^2+^ + PO_4_^3−^ → Fe_3_(PO_4_)_2_

9 Fe^3+^ + PO_4_^3−^ → FePO_4_

10 Fe(OH)_3(s)_ + PO_4_^3−^ → Fe(OH)_3_

<svg xmlns="http://www.w3.org/2000/svg" version="1.0" width="23.636364pt" height="16.000000pt" viewBox="0 0 23.636364 16.000000" preserveAspectRatio="xMidYMid meet"><metadata>
Created by potrace 1.16, written by Peter Selinger 2001-2019
</metadata><g transform="translate(1.000000,15.000000) scale(0.015909,-0.015909)" fill="currentColor" stroke="none"><path d="M80 600 l0 -40 600 0 600 0 0 40 0 40 -600 0 -600 0 0 -40z M80 440 l0 -40 600 0 600 0 0 40 0 40 -600 0 -600 0 0 -40z M80 280 l0 -40 600 0 600 0 0 40 0 40 -600 0 -600 0 0 -40z"/></g></svg>

PO_4_

11 FeOH + PO_4_^3−^ + H^+^ → FePO_4_^2−^ + H_2_O

## 4. Conclusions

In this work, the simple coupling of oxidant and ZVI was used to activate the surface reactivity of ZVI, promote the corrosion of ZVI and the continuous activation of the passivation layer, so as to improve P removal. Due to the different oxidation properties of the oxidants themselves, the corrosion situation on ZVI also varies. In the batch study, the H_2_O_2_/ZVI, NaClO/ZVI and Na_2_S_2_O_8_/ZVI systems increased by 12.9%, 83.7% and 1.3 times, respectively, and this promoting effect was enhanced with increasing oxidant dosage. Five batch cycle tests also proved that oxidants can alleviate the impedance problem of the ZVI passivation layer. In addition, ZVI alone and NaClO/ZVI systems were selected for long-term column research. The removal capacity and operating life of the NaClO/ZVI system (23.7 mg g^−1^, 4080 PV) are far greater than the ZVI alone system (2.6 mg g^−1^, 970 PV). It follows that oxidants are able to readily solve the passivation problem during ZVI corrosion and are of great significance for ZVI to remove P from water.

## Conflicts of interest

There are no conflicts to declare.

## Supplementary Material
